# Placental immune editing switch (PIES): learning about immunomodulatory pathways from a unique case report

**DOI:** 10.18632/oncotarget.13306

**Published:** 2016-11-11

**Authors:** Miguel H. Bronchud, Francesc Tresserra, Wenjie Xu, Sarah Warren, Maite Cusido, Bernat Zantop, Ana Claudia Zenclussen, Alessandra Cesano

**Affiliations:** ^1^ Institut Bellmunt Oncologia, Hospital Universitario Dexeus. Grupo Quirón Salud, Barcelona, Spain; ^2^ Servei Anatomia Patològica, Hospital Universitario Dexeus. Grupo Quirón Salud, Barcelona, Spain; ^3^ Nanostring Technologies, Immune Oncology, Seattle, WA, USA; ^4^ Ginecologia Oncològica, Hospital Universitario Dexeus. Grupo Quirón Salud, Barcelona, Spain; ^5^ Servei Obstetricia i Neonatologia, Hospital Universitario Dexeus. Grupo Quirón Salud, Barcelona, Spain; ^6^ Experimental Obstetrics and Gynecology, Medical Faculty, Otto-von-Guericke University Magdeburg, Magdeburg, Germany

**Keywords:** materno-fetal tolerance, cancer microenvironment, placental microenvironment, immune vigilance, carcinogenesis

## Abstract

The hypothesis of this work is that, in order to escape the natural immune surveillance mechanisms, cancer cells and the surrounding microenvironment might express ectopically genes that are physiologically present in the placenta to mediate fetal immune-tolerance. These natural “placental immune-editing switch” mechanisms (PIES) may represent the result of millions of years of mammalian evolution developed to allow materno-fetal tolerance. Here, we introduce genes of the immune regulatory pathways that are either similarly over- or under-expressed in tumor *vs* normal tissue. Our analysis was carried out in primary breast cancer with metastatic homolateral axillary lymph nodes as well as placenta tissue (both uterine decidual tissue and term placenta tissue) from a pregnant woman. Gene expression profiling of paired non-self and self tissues (i.e. placenta/uterus; breast cancer/normal breast tissue; metastatic lymphnode/normal lymphnode tissue) was performed using the PanCancer Immune gene panel, a 770 Nanostring gene expression panel. Our findings reveal overlapping in specific immune gene expression in placenta and cancer tissue, suggesting that these genes might play an important role in maintaining immune tolerance both physiologically (in the placenta) and pathologically (in the cancer setting).

## INTRODUCTION

Cancer microenvironment has been recognized to be a crucial determinant of cancer cells behavior through both positive and negative effects on tumor growth. In clinical detectable tumors, the microenvironment is usually immune suppressive, and strategies that inactivate molecules or mechanisms involved in the induction and maintenance of T-cell tolerance offer great therapeutic promise [1, 2].

In the past decade, several therapeutic approaches have entered the clinical setting with remarkable success, including CTLA-4 blockade with humanized monoclonal antibodies (Mabs) [3–5], which affects mainly the immune central tolerance and blockade of the PD-L1/PD-1 axis, which regulates negatively TCR signals and affects mainly peripheral T-cell tolerance [6–8].

To date, three humanized Mabs (pembrolizumab, nivolumab and atezolumab) blocking the PD-1/PD-L1 pathway have been approved in USA and outside USA in many clinical indications including metastatic malignant melanoma, renal cell cancer, urothelial tumors, lung and head and neck cancers.

Increasing understanding of cellular and molecular tumor immunology has enabled the identification of new and innovative ways to manipulate the immune response to cancer and has opened the door to multiple combination treatments, including combinations between different types of immunotherapies as well as combination of immunotherapy with standard cytotoxic and targeted therapies (a useful and wide review on the subject of new cancer immune therapies was presented at ASCO 2016 in Chicago,https://www.asco.org/research-progress/reports-studies/clinical-cancer-advances#/advance-year-cancer-immunotherapy).

A typical immune response in a health individual originates with dendritic cells (DC), which are responsible for initiating all antigen-specific immune responses. As such, they can be considered the master regulators of the immune response. These cells activate T cells by a complex molecular mechanism by which the peptides are presented by the MHC molecules and are recognized by T-cell receptors, and these T cells can differentiate into various effectors including cytotoxic and helper T cells.

The immune response can be modulated at the molecular level, by soluble factors, like cytokines, and at the cellular level, by direct cell-to-cell interactions. In advanced human cancers, suppressive rather than inflammatory immune responses seem to govern.

Therefore, in order to boost immunosurveillance it is crucial to block immunotolerance. The mechanisms leading to tumor tolerance are however not fully understood. In our eyes, not enough attention has been paid to the human placenta as the key to understand immune tolerance mechanisms in cancer. It has been known for a long time [9] that pregnant women can normally tolerate millions of fetal circulating cells in the bloodstream as well as the fetus with its placenta without leading to their immune rejection. Additionally, as soon as the baby is born, the mother would immediately reject tissues containing fetal cells. This suggests that the placenta is the key organ orchestrating tolerance. According to the “placental immune-editing switch” (PIES) hypothesis some cancer cells and/or their microenvironment are capable of inappropriately activating this “switch”, typical of the human placenta, to block immune rejection and promote immune tolerance.

In the past two decades several interesting mechanisms of immune modulation by the placenta have been described although a complete understanding of the process is still missing [10–15]. Mechanisms responsible for fetomaternal tolerance are probably multiple and diverse [9–16] with many different players [17–21] ranging from the expression of non classical MHC molecules (like HLA-G, HLA-E or HLA-C) by trophoblast cells, tryptophan catabolism by the enzyme IDO (Indoleamine 2,3-dioxygenase), T cell apoptosis and regulatory T-cells (Tregs), the inhibitory costimulatory molecules like programmed cell death ligand (PDL)1, the Fas ligand pathways, complement system and microvesicles released by the placenta into the circulation of the mother, and even thymic stromal lymphopoietin from trophoblasts which induces dendritic cell-mediated regulatory Th2 bias in the deciduas during early gestation in humans [21]. Epigenetic models of regulation have also been implicated in the development and maintenance of feto-maternal tolerance, in particular with reference to the selective down-regulation of several MHC paternal antigens once the fetal cells approach or cross the human placenta [10].

The potential similarities between the mechanisms involved in feto-maternal and tumor-associated immunologic tolerance are intriguing, and can now be interrogated by using new genomic and epigenomic technologies.

In this study, building on the unique opportunity of availability of non-self and self tissues from a single pregnant patient with breast cancer we sought to identify genes whose expression overlapped between placenta and malignant tissue. The identification of these genes that might play an important role in maintaining immune tolerance both physiologically (in the placenta) and pathologically (in the cancer setting) will help to design new cancer strategies in the future.

## RESULTS

### Histological results of collected tissues

**Figure 1:**
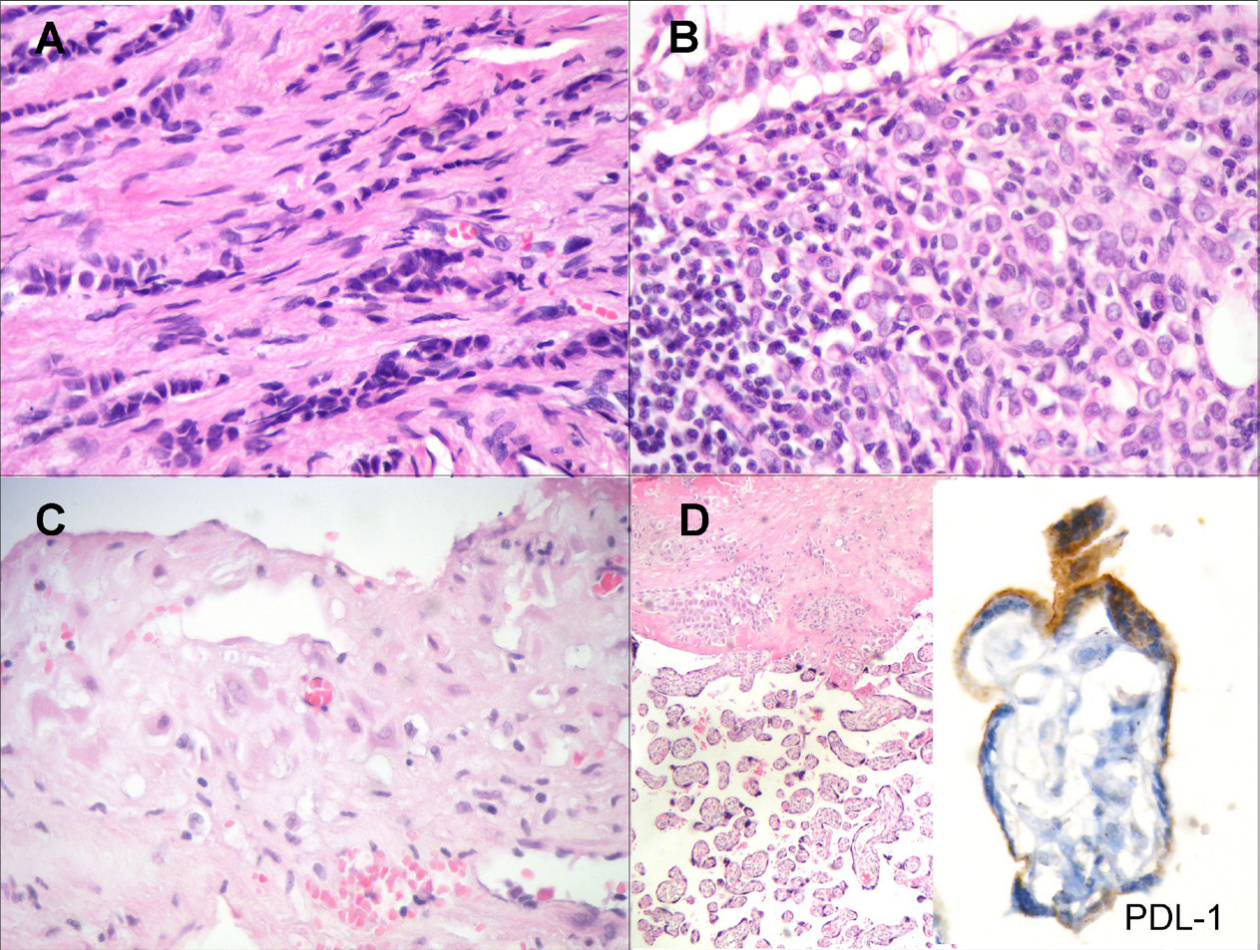
**A.** Lobular invasive carcinoma of the breast with epithelial single-cell files infiltrating the stroma. There is a mild nuclear pleomorfism (H&amp;E x 400). **B.** Invasion of the lymph node parenchyma by an epithelial proliferation of tumor cells with characteristics similar to the primary breast carcinoma (H&amp;E x 400). **C.** Decidual transformation of endometrial mucosa with epithelioid appearance of stromal cells (H&amp;E x 400). **D.** Placental villi with normal architecture (Left) (H&amp;E x40). PDL-1 immunohistochemical stain was positive in some trophoblastic cells of the surface lining (Right) (x 1000)

Histologically, the breast carcinoma was classified as LBC, with some areas of *in situ* component, stage pT2 pN2a Mo (5/15 axillary lymph nodes with metastatic disease), and with immunohistochemistry characteristics of Luminal A subtype (ER60%+, PR50%+, HER-2 negative and Ki67 5% positivity). There was a mild nuclear pleomorfism (Figure 1A). Invasion of the lymph node parenchyma by an epithelial proliferation of tumor cells with similar characteristics to those of the primary breast carcinoma was observed in 5/15 of the surgically removed nodes (Figure 1B). The uterus histologically showed physiologic decidual transformation of endometrial mucosa with characteristic epithelioid appearance of stromal cells (as shown in Figure 1C); and the placenta had villi with normal architecture Figure 1D Left (H&E x40). Finally, PDL-1 immunohistochemical stain was also positive in some trophoblastic cells of the surface lining (Figure 1D Right) (x 1000).

### Tissue specific gene expression

**Figure 2:**
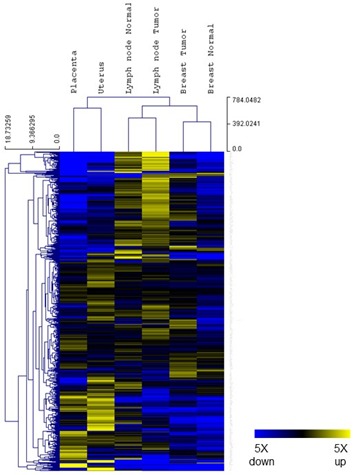
Tissue gene expression. Relative expression levels for 583 genes from the PanCancer Immune profiling panel (normalized to the mean value of the 6 different tissue samples) were shown in a heat map. Blue indicates expression level below mean and yellow indicates expression above mean. Both genes and samples were grouped by unsupervised clustering.

The gene expression profiling analysis was designed to assess whether there were common patterns of immune gene expression shared between placenta and cancer tissue. Six tissue samples were tested from a single patient: placenta and uterus (representing non-self *vs*. self-tissue from pregnancy), breast cancer and normal breast tissue, as well as tumor bearing lymph node and non-involved lymph node. Gene expression data were gathered on the nCounter system and analyzed using nSolver3.0 software (NanoString Technologies). All six samples passed data QC and were normalized by housekeeping genes (details described in Materials and Methods). Raw and normalized data are provided in supplemental Table 1. Housekeeping genes and low expressed genes were excluded from further analysis, as described in MM, leaving 583 genes for analysis. An unsupervised clustering of gene expression ratios between each individual sample and the mean of all six samples was first performed. As shown in Figure 2, placenta and uterus, breast tissues (tumor and normal) and lymph node tissues (tumor and normal) formed their own cluster, suggesting that tissue specific gene expression patterns were well preserved during experimental procedures and that tissue specific expression pattern overshadows gene expression differences within each tissue type.

### Gene expression within each tissue type

**Figure 3:**
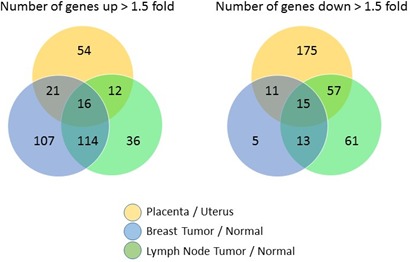
Relative expression levels of paired non-self *versus* self tissue for 583 genes from the PanCancer Immune profiling panel are shown in a Venn Diagram to highlight the number of commonly and uniquely regulated genes among the three different tissue types

We then analyzed differential expression within each tissue type (i.e. matching for the analysis placenta with uterus, breast cancer with normal breast tissue and node positive with node negative tissue). Among the 583 genes analyzed, 103 genes were upregulated > 1.5 fold in placenta *versus* uterus, while 258 genes were downregulated at least 1.5 fold. Using the same cut-off, 258 genes were upregulated and 44 genes downregulated in breast tumor *versus* normal breast tissue, and 178 genes were upregulated while 146 genes downregulated in tumor bearing lymph node *versus* non-involved lymph node. Figure 3 summarizes overlapping and unique differentially expressed genes among the three tissue types. Complete sets of gene expression ratios are available in supplemental Table 2.

### Common expression patterns

**Figure 4:**
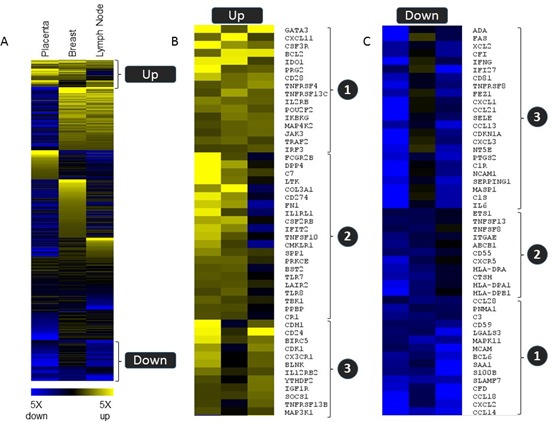
Common gene expression patterns regulation in three tissue types. Relative expression levels for non-self *versus* self tissue for 583 genes from the PanCancer Immune profiling panel are shown in a heat map (supervised clustering) to demonstrate subsets of genes that were commonly regulated in all three tissue types (group 1) or that were similarly regulated in placenta and one of the tumor tissue samples. (group 2 and group 3). Blue indicates down regulation in the non-self tissue, and yellow indicates upregulation.

To help visualize common gene expression patterns i.e. genes consistently differentially expressed in non-self tissue *versus* the corresponding self tissue, a heat map based on gene expression ratios within the three tissue types was generated (Figure 4A). Genes that are up- or downregulated 1.5 fold or more in at least two of the three tissue types are represented in Figure 4. Sixteen genes were upregulated and fifteen genes were downregulated in all three tissue types (Figure 4B/4C, group 1). The fold changes of these commonly regulated genes are shown in Table 1 (upregulated genes) and Table 2 (downregulated genes). Group 2 (Figure 4B/4C) contain genes commonly regulated in placenta *versus* uterus and breast tumor *versus* normal, and group 3 consist of genes commonly regulated in placenta *versus* uterus and metastatic *versus* normal lymph node tissues.

**Table 1:** Genes over-expressed in malignant tissues (breast and metastatic LN), to levels similar to placental and decidual tissues

	Placenta/Uterus	Breast tumor/normal	Lymph node tumor/normal
**BCL2**	1.93	4.45	4.30
**CD28**	3.41	1.99	2.33
**CSF3R**	10.69	2.56	2.00
**CXCL11**	1.97	16.54	1.53
**GATA3**	6.57	1.76	24.97
**IDO1**	4.52	4.57	1.53
**IKBKG**	1.52	2.13	1.92
**IL2RB**	2.26	1.93	1.85
**IRF3**	1.54	1.56	1.74
**JAK3**	1.50	1.84	1.94
**MAP4K2**	1.60	1.84	1.95
**POU2F2**	2.03	2.24	1.68
**PRG2**	5.33	1.72	1.84
**TNFRSF13C**	1.61	2.79	1.80
**TNFRSF4**	1.70	2.00	2.53
**TRAF2**	1.78	1.93	1.53

**Table 2:** Genes under-expressed in malignant tissues (breast and metastatic LN), to levels similar to placental and decidual tissues

	Placenta/Uterus	Breast tumor/normal	Lymph node tumor/normal
**BCL6**	−2.57	−1.85	−3.64
**C3**	−2.29	−1.98	−1.64
**CCL14**	−169.11	−3.09	−4.34
**CCL18**	−5.46	−1.66	−8.33
**CCL28**	−1.56	−2.17	−1.86
**CD59**	−1.80	−1.81	−2.36
**CFD**	−3.62	−1.77	−15.18
**CXCL2**	−37.99	−1.66	−3.83
**LGALS3**	−1.94	−1.72	−4.40
**MAPK11**	−1.85	−3.04	−2.49
**MCAM**	−1.71	−2.16	−6.36
**PNMA1**	−2.71	−1.59	−1.73
**S100B**	−2.19	−1.98	−16.68
**SAA1**	−2.32	−1.53	−25.50
**SLAMF7**	−3.41	−2.69	−2.89

## DISCUSSION

The survival of the fetus within the maternal womb has fascinated, and still does, researchers for years. Normally, the immune system would recognize foreign antigens and destroy them. For pregnancy to be successful, the paternal antigens expressed by the placenta and the fetus need to be recognized and tolerated by the maternal immune system. Although many studies address this intriguing question, the mechanisms allowing pregnancy tolerance are still not fully understood. In this study, we had the unique opportunity to investigate gene expression in tissue samples collected from a patient who developed breast cancer during the pregnancy. This study was designed to assess whether there were common patterns of immune gene expression shared between placenta and uterus (representing non-self *versus* self tissue from pregnancy), breast cancer *versus* normal breast tissue, and tumor bearing lymph node *vs* non-involved lymph node. The hypothesis of the study was that the common requirement for persistent immune evasion in all tissue settings could result in common patterns of immune gene regulation.

IDO1, which has been extensively characterized as an inhibitor of immune responses in the tumor microenvironment, was observed to be upregulated in all the three non-self tissues. IDO1 is an enzyme which catalyzes the essential amino acid tryptophan into its metabolite kynurenine [25]. Depletion of tryptophan from the local tumor environment has been shown to inhibit proliferation and activity of T cells, profoundly limiting anti-tumor immune responses [25–29]. Based on these observations, IDO1 small molecule antagonists are actively being investigated in the clinic in a variety of tumors, alone or in combination with immune checkpoint blockade or cancer vaccines [25–29]. In the context of pregnancy, IDO1 has shown to be expressed by fetal and placental tissue and is believed to participate, at least in part, in rejection evasion by the maternal immune system [30], although likely in conjunction with other mechanisms, since IDO1 knock out (KO) mice have normal pregnancies and do not show autoimmune symptoms [31, 32].

In addition to IDO1, PD-L1 (CD274) was also upregulated in placenta and breast tumor as compared to uterus and normal breast tissue. A similar upregulation of PDL1 was not observed in the lymph node bearing metastatic breast tumor, although this may be due to the existence of other immune regulatory pathways in the lymph node or be attributed to the different tissue types being characterized (metastasized breast tumor *vs* healthy lymphoid tissue), or to the presence of activated leukocytes within the lymph node in response to antigen draining from the distal tumor, which can express PD-L1 in some conditions and therefore mask the final result [33, 34]. PD-L1 is also expressed by placental mesenchymal stem cells during pregnancy, which further lends support to the discovery method used in this study [35]. Of note, PD-1, the receptor for PD-L1 is expressed by regulatory T cells. In our clinical-pathological case, FOXP3 (which is the hall-mark of regulatory T cells) is upregulated in both breast cancer and metastatic lymph node (2.72 and 4.15 fold up comparing to normal tissue, respectively), but its expression level is insignificantly changed in uterus when compared to normal breast or lymph node tissue (1.23 and 1.26 fold, respectively), and its expression level is actually down-regulated in placenta compared to normal breast or lymph node tissue (0.54 and 0.56 fold, respectively). However, the raw counts for FOXP3 were very low, all in the 20-30 counts range, which is close to the assay detection limit except for metastatic lymph node where 144 counts were measured, so that the only solid conclusion we can draw from this single case is that FOXP3 is upregulated in the metastatic lymph node.

By contrast, both TGF-β1 and 2 were significantly increased in both malignant tissues as well as in uterus and placenta. TGF-β is a multifunctional cytokine belonging to the transforming growth factor superfamily that includes three different isoforms (TGF β 1, TGF β 2, TGF β 3) and many other signaling proteins produced by all white blood cell lineages. After the binding of TGF-β, the type 2 receptor kinase phosphorylates and activates the type 1 receptor kinase that activates a signaling cascade. This leads to the activation of different downstream substrates and regulatory proteins, inducing transcription of different target genes that function in differentiation, chemotaxis, proliferation, and activation of many immune cells; importantly, the final net effects are context dependent. Specifically, TGF-β1 alone stimulates the expression of Foxp3 and regulatory Tcell differentiation from activated T helper cells and has mainly inhibitory effects on B lymphocytes. In addition, TGF-β has been shown to downregulate inflammatory cytokine production in monocytes and macrophages, likely by the inhibition of NF-κB pathway. The importance of TGF β in pregnancy is highlighted by the fact that TGF β KO animals die in uterus. Additionally, it has been shown that TGF β present in seminal vesicle fluid is able to promote the conversion of vaginal T cells into regulatory T cell in murine models, which is a first step in promoting pregnancy tolerance [36].

Beyond IDO1, PD-L1 and TGFG β as prototypical examples of well-known immune inhibiting proteins identified in this study, few other immune modulating proteins were identified as differentially regulated in the non-self tissues *versus* the normal counterpart. Specifically, there was a rise in genes associated with NKFB signaling, including IKBKA, MAP4K2, JAK3, TRAF2, and IRF2. This association was restricted to upregulated genes, as genes significantly downregulated in nonself tissue *vs* self did not include genes associated with regulation of NF-kB signaling. Genes significantly downregulated in two or more tissues include IFNγ and the IFNγ regulated genes IFI27 and CXCL1 (IP-10). This observation would suggest an absence of activated T cells from the local environment. However, a number of T cell associated genes were upregulated in the non-self tissue, including the costimulatory molecules CD28 and TNFRSF4 (OX40LR), IL2RB, GATA3, and the IFNγ induced chemokine CXCL11. Since the immune system works in a network fashion, the clinical outcome of any immune response is better represented by the net result of positive and negative modulators than by the absolute values of each of the single components, thus the importance of using a multi-plexed approach.

Interestingly, 4 of the 15 genes whose expression was commonly decreased in the non-self paired tissues compared to the respective self counterpart belong to the chemokines family suggesting that modulation of mechanisms involved in immune cell trafficking could be an important contributor to peripheral tolerance mechanisms. For instance, CCL28 regulates the chemotaxis of cells that express the chemokine receptors CCR3 and CCR10 such as eosinophils, basophils and some T cell subsets.

Several genes associated with myeloid derived suppressor cells (MDSC) activity (including TNFAIP3 and XCL2), were also observed to be upregulated in both the breast tumor and the placenta tissue. MDSCs are a heterogeneous group of myeloid cells that suppress both innate and adaptive immune responses through multiple mechanisms. In recent years, much of our knowledge of the function of MDSCs has come from cancer studies [37–39]. However, few recent studies have begun to characterize MDSCs in feto-maternal immune cross-talk and current data show that MDSCs accumulate at the fetal-maternal interface in healthy pregnancies and can play key immune regulatory rules [40–42].

Finally, the surface antigen CD319 (SLAMF7) is a robust marker of plasma cells. The observation of decreased gene transcripts in non-self tissues would therefore suggest the participation of the B cell compartment of the immune system in peripheral tolerance [43]. Indeed, IL-10 producing B cells emerge as novel components of immune tolerance in the context of autoimmune responses and pregnancy [44, 45].

The ultimate purpose of a discovery effort such as the one presented here is to identify novel genes that are commonly upregulated or downregulated in all non-self tissues and in this respect this single patient study provided a hint of the list of gene candidates although the data cannot yet be tied up into main PIES specific immune regulatory pathway(s).

Evaluation of additional samples will increase the power of these preliminary observations and may reveal additional genes or pathways that merit further investigation.

## MATERIALS AND METHODS

### Case description

In the year 2000, a 32 years old pregnant woman with a palpable breast lump underwent delivery of non-identical twins by C-section at week 39 of a normally developing pregnancy. A total hysterectomy was performed during the C-section to control for profuse bleeding caused by attempting manual separation of the complex membranous placenta with velamentous umbilical cord insertion. Following histological confirmation of the malignant nature of the breast nodule, the patient underwent right mastectomy and axillary lymphadenectomy (with removal of 15 lymph nodes). The tumor was reported as invasive lobular breast carcinoma (LBC) stage pT2 pN2a Mo (5/15 axillary lymph nodes with metastatic disease), with immunohistochemistry (IHC) compatible with “Luminal A subtype” (ER+,PR+,HER-2 negative and Ki67 5% positivity) and histological evidence of vascular and lymphatic invasion by cancer cells and some lobular carcinoma *in situ* as background component. The patient received standard of care adjuvant chemotherapy, loco regional radiotherapy and adjuvant hormone therapy (tamoxifen for five years) and was followed for over ten years without evidence of loco-regional or distant recurrence.

### Tissue samples

The original paraffin embedded samples of the mastectomy and axillary lymph node dissection tissue as well as of the placenta and uterus were recovered and gene expression profiling analysis using the PanCancer Immune Profiling panel on the nCounter System (Nanostring Technologies, Seattle) was performed using these tissue samples.

Specifically, six types of tissue samples obtained from the patient were analyzed:

1. Uterine decidual tissue (the uterine tissue was microdissected in paraffin to enrich decidual tissues)
2. Placenta
3. Breast tumor:
4. Metastatic axillary lymph nodes
5. Normal breast tissue from the mastectomy specimen
6. Normal axyllary lymph nodes from lymphadenectomy

### Histopathology methods

Representative tissue from breast tumor, axillary lymph node, decidua and placenta were fixed in 10% formalin buffered at room temperature for 24 hours. Samples were paraffin embedded and stained according hematoxylin-eosin standard techniques. Additional sections of breast cancer were stained with immune-histochemical procedures for detection of estrogen receptor (Clone ER-SP1. RTU, Ventana/Roche. Tucson, Arizona), progesterone receptor (Clone PR-1E2. RTU, Ventana/Roche. Tucson, Arizona), p53 (Clone D07. RTU, Ventana/Roche. Tucson, Arizona), HER2 (Clone 4B5, RTU, Pathway, Ventana/Roche. Tucson, Arizona) and Ki67 (Clone 30-9. RTU, Ventana/Roche. Tucson, Arizona). In placental samples PDL1 expression was also measured by immunohistochemistry (IHC) (Clone CD274. Dilution 1/200. Novus Biologicals. Littleton CO).

### Gene expression profiling

Gene expression profiling was performed using the PanCancer Immune Profiling panel on nCounter system (Nanostring Technologies, Inc., Seattle, WA).

The *nCounter PanCancer Immune Profiling Panel* is a unique 770-plex gene expression panel designed to measure the human immune response in both solid and liquid cancer types. The list of the gene measured by this panel is provided in supplemental Table 1. The assay is run on the *nCounter Analysis System* (Nanostring Technologies, Inc.), an automated system which received 510(k) clearance from the FDA for use with the Prosigna Breast Cancer Prognostic Gene Signature Assay [22, 23]. The *nCounter Analysis System* is based on a digital color-coded barcode technology which allows for direct multiplexed measurement of gene expression from low amount of mRNA (25 to 300 ng) without need for amplification. At a very high level, the assay includes three main steps:

#### Hybridization

specific pairs of a “capture” and a “reporter” probe are provided for each gene of interest, allowing up to 800 genes to be multiplexed, and their mRNA transcript levels measured, in a single experiment, for each sample. The “reporter” probe carries the signal, and the “capture” probe allows the complex to be immobilized for data collection.

#### Purification and immobilization

after hybridization, samples are transferred to the nCounter Prep Station where excess probes are removed and probe/target complexes are bound, immobilized, and aligned on the *nCounter Cartridge*.

#### Counting and analysis

sample cartridges are placed in the *nCounter Digital Analyzer* for data collection. Barcodes are counted and tabulated for each target by the *nCounter Digital Analyzer*. *The raw data are then imported into the analysis software (nSolver3.0)* that automatically performs QC, normalization, data analysis and creates multi-page reports with the options of performing advanced analyses including pathway applications.

The time from sample lysates to data results is two days and because the process is highly automated the hands-on time (and therefore chances for human errors) is limited (25 min per 12 samples). In this study the assay was performed by a Contract Research Organization (IZASA Spain).

The *nCounter PanCancer Immune Profiling Panel* provides an “off the shelf” multiplexed gene expression panel designed to quantify 770 genes that fall into four functional categories [23]:

**Unique transcripts which allow for the identification and quantification of 24 different immune cell types**

**Transcripts measuring specific immunological functions** (innate, adaptive)

**Transcripts for tumor-shared antigens**, such as cancer-testis (CT) antigens.

**Housekeeping genes** that facilitate sample-to-sample normalization.

### Sample collection and RNA isolation

High Pure FFPET RNA Isolation Kit (ROCHE Diagnostics GmbH) was used for the isolation and purification of total RNA from formalin-fixed, paraffin-embedded tissue samples. The quality of RNA from tissue samples was suitable for testing according to proprietary guidelines (https://lifescience.roche.com/shop/products/high-pure-ffpet-rna-isolation-kit).

### NanoString ncounter profiling

Approximately 300 ng of total RNA isolated from FFPE slices for each of the six tissue samples were mixed with reporter and capture probes from the PanCancer Immune Profiling panel (NanoString Technologies) and hybridized for 18 hours at 65°C. The samples were processed on nCounter Prep Station using the high sensitivity protocol, followed by imaging and counting on the nCounter digital analyzer (NanoString Technologies, following manufacturer's protocols) [24].

### NanoString data analysis

RCC files from NanoString digital analyzer were imported into nSolver3.0 (NanoString Technologies) and were checked for data quality using default QC settings. All samples passed QC. Specifically, > 95% of FOV were successfully counted with binding densities between 0.2 and 0.5, and positive controls generated expected counts and demonstrated good linearity. Background subtraction was carried out by subtracting the mean value of the eight negative control ERCC sequences from the raw counts of all endogenous genes. Data sets were normalized using the geometric mean of 25 housekeeper genes with the lowest CV%. Because there is a single sample from each tissue type (N = 1) in this case study, we decided to use only high confidence data points for analysis. Genes with mean normalized counts less than 30 (2 standard deviations from the mean of background controls) were excluded from further analysis (147 genes excluded). Gene expression ratios were calculated by dividing the normalized counts of one sample to that of another sample or to the mean value of all six samples. Data generated by the nCounter system are highly reproducible and can distinguish as low as 1.2 fold differences in target abundance levels. Given the limitation of sample size in this study, we decided to use 1.5 fold change as the threshold for differential expression. Heat maps in Figures 1 and 3 were generated using Multiexperimental Viewer 4.9.0.

## SUPPLEMENTARY FIGURES AND TABLES





## References

[1] Steinman RM, Hawiger D, Nussenzweig MC (2003). Tolerogenic dendritic cells. Annu Rev Immunol.

[2] Steinman RM, Hawiger D, Liu K, Bonifaz L, Bonnyay D, Mahnke K, Iyoda T, Ravetch J, Dhodapkar M, Inaba K, Nussenzweig M (2003). Dendritic cell function *in vivo* during the steady state: a role in peripheral tolerance. Ann N Y Acad Sci.

[3] Korman AJ, Peggs KS, Allison JP (2006). Checkpoint blockade in cancer immunotherapy. Adv Immunol.

[4] Dranoff G (2005). CTLA-4 blockade: unveiling immune regulation. J Clin Oncol Off J Am Soc Clin Oncol.

[5] Alegre M-L, Fallarino F (2006). Mechanisms of CTLA-4-Ig in tolerance induction. Curr Pharm Des.

[6] Blank C, Gajewski TF, Mackensen A (2005). Interaction of PD-L1 on tumor cells with PD-1 on tumor-specific T cells as a mechanism of immune evasion: implications for tumor immunotherapy. Cancer Immunol Immunother CII.

[7] Keir ME, Liang SC, Guleria I, Latchman YE, Qipo A, Albacker LA, Koulmanda M, Freeman GJ, Sayegh MH, Sharpe AH (2006). Tissue expression of PD-L1 mediates peripheral T cell tolerance. J Exp Med.

[8] Cardoso A, Bronchud MH, Foote MA, Giaccone G, Olopade O, Workman P (2008). Harnessing the power of immunity to battle cancer: much ado about nothing or all's well that ends well?. Principles of Molecular Oncology.

[9] Medawar PB (1953). Some immunological and endocrinological problems raised by the evolution of viviparity in vertebrates. Soc Exp Biol.

[10] Guleria I, Sayegh MH (2007). Maternal acceptance of the fetus: true human tolerance. J Immunol Baltim Md 1950.

[11] Kaufman KA, Bowen JA, Tsai AF, Bluestone JA, Hunt JS, Ober C (1999). The CTLA-4 gene is expressed in placental fibroblasts. Mol Hum Reprod.

[12] Wang X, Ma Z, Hong Y, Lu P, Lin Q (2006). Expression of CD28 and cytotoxic T lymphocyte antigen 4 at the maternal-fetal interface in women with unexplained pregnancy loss. Int J Gynaecol Obstet Off Organ Int Fed Gynaecol Obstet.

[13] Petroff MG, Chen L, Phillips TA, Azzola D, Sedlmayr P, Hunt JS (2003). B7 family molecules are favorably positioned at the human maternal-fetal interface. Biol Reprod.

[14] Hunt JS, Vassmer D, Ferguson TA, Miller L (1997). Fas ligand is positioned in mouse uterus and placenta to prevent trafficking of activated leukocytes between the mother and the conceptus. J Immunol Baltim Md 1950.

[15] Guleria I, Khosroshahi A, Ansari MJ, Habicht A, Azuma M, Yagita H, Noelle RJ, Coyle A, Mellor AL, Khoury SJ, Sayegh MH (2005). A critical role for the programmed death ligand 1 in fetomaternal tolerance. J Exp Med.

[16] Dunn GP, Bruce AT, Ikeda H, Old LJ, Schreiber RD (2002). Cancer immunoediting: from immunosurveillance to tumor escape. Nat Immunol.

[17] Munoz-Suano A, Hamilton AB, Betz AG (2011). Gimme shelter: the immune system during pregnancy. Immunol Rev.

[18] Tilburgs T, Scherjon SA, Claas FHJ (2010). Major histocompatibility complex (MHC)-mediated immune regulation of decidual leukocytes at the fetal-maternal interface. J Reprod Immunol.

[19] Gobert M, Lafaille JJ (2012). Maternal-fetal immune tolerance, block by block. Cell.

[20] Leber A, Teles A, Zenclussen AC (2010). Regulatory T cells and their role in pregnancy. Am J Reprod Immunol N Y N 1989.

[21] Guo P-F, Du M-R, Wu H-X, Lin Y, Jin L-P, Li D-J (2010). Thymic stromal lymphopoietin from trophoblasts induces dendritic cell-mediated regulatory TH2 bias in the decidua during early gestation in humans. Blood.

[22] Geiss GK, Bumgarner RE, Birditt B, Dahl T, Dowidar N, Dunaway DL, Perry Fell H, Ferree S, George RD, Grogan T, James JJ, Maysuria M, Mitton JD (2008). Direct multiplexed measurement of gene expression with color-coded probe pairs. Nat Biotechnol.

[23] Cesano A (2015). nCounter(^®^) PanCancer Immune Profiling Panel (NanoString Technologies, Inc., Seattle, WA). J Immunother Cancer.

[24] nSolver with Nanostring PanCancer Immune Kit http://wwwnanostringcom/applications/technology.

[25] van Baren N, Van den Eynde BJ (2015). Tumoral Immune Resistance Mediated by Enzymes That Degrade Tryptophan. Cancer Immunol Res.

[26] Epacadostat Before Surgery in Treating Patients With Newly Diagnosed Stage III-IV Epithelial Ovarian, Fallopian Tube, or Primary Peritoneal Cancer - Full Text View - ClinicalTrials.gov [Internet] https://clinicaltrialsgov/ct2/show/NCT02042430?term=ido1&rank=3.

[27] DEC-205/NY-ESO-1 Fusion Protein CDX-1401, Poly ICLC, and IDO1 Inhibitor INCB024360 in Treating Patients With Ovarian, Fallopian Tube, or Primary Peritoneal Cancer in Remission - Full Text View - ClinicalTrials.gov [Internet] https://clinicaltrialsgov/ct2/show/NCT02166905?term=ido1&rank=2.

[28] IDO Inhibitor in Advanced Solid Tumors - Full Text View - ClinicalTrials.gov [Internet] https://clinicaltrialsgov/ct2/show/NCT02048709?term=ido1&rank=10.

[29] A Phase 1/2 Randomized, Blinded, Placebo Controlled Study of Ipilimumab in Combination With INCB024360 or Placebo in Subjects With Unresectable or Metastatic Melanoma - Full Text View - ClinicalTrials.gov [Internet] https://clinicaltrialsgov/ct2/show/NCT01604889?term=ido1&rank=9.

[30] Sedlmayr P, Blaschitz A, Stocker R (2014). The role of placental tryptophan catabolism. Front Immunol.

[31] Munn DH, Zhou M, Attwood JT, Bondarev I, Conway SJ, Marshall B, Brown C, Mellor AL (1998). Prevention of allogeneic fetal rejection by tryptophan catabolism. Science.

[32] Munn DH (2006). Indoleamine 2,3-dioxygenase, tumor-induced tolerance and counter-regulation. Curr Opin Immunol.

[33] Bertucci F, Finetti P, Colpaert C, Mamessier E, Parizel M, Dirix L, Viens P, Birnbaum D, van Laere S (2015). PDL1 expression in inflammatory breast cancer is frequent and predicts for the pathological response to chemotherapy. Oncotarget.

[34] Haile ST, Bosch JJ, Agu NI, Zeender AM, Somasundaram P, Srivastava MK, Britting S, Wolf JB, Ksander BR, Ostrand-Rosenberg S (2011). Tumor cell programmed death ligand 1-mediated T cell suppression is overcome by coexpression of CD80. J Immunol Baltim Md 1950.

[35] Abumaree MH, Al Jumah MA, Kalionis B, Jawdat D, Al Khaldi A, Abomaray FM, Fatani AS, Chamley LW, Knawy BA (2013). Human placental mesenchymal stem cells (pMSCs) play a role as immune suppressive cells by shifting macrophage differentiation from inflammatory M1 to anti-inflammatory M2 macrophages. Stem Cell Rev.

[36] Schumacher A, Heinze K, Witte J, Poloski E, Linzke N, Woidacki K, Zenclussen AC (2013). Human chorionic gonadotropin as a central regulator of pregnancy immune tolerance. J Immunol Baltim Md 1950.

[37] Gabrilovich DI, Nagaraj S (2009). Myeloid-derived suppressor cells as regulators of the immune system. Nat Rev Immunol.

[38] Chun E, Lavoie S, Michaud M, Gallini CA, Kim J, Soucy G, Odze R, Glickman JN, Garrett WS (2015). CCL2 Promotes Colorectal Carcinogenesis by Enhancing Polymorphonuclear Myeloid-Derived Suppressor Cell Population and Function. Cell Rep.

[39] Marvel D, Gabrilovich DI (2015). Myeloid-derived suppressor cells in the tumor microenvironment: expect the unexpected. J Clin Invest.

[40] Zhao A-M, Xu H-J, Kang X-M, Zhao A-M, Lu L-M (2016). New insights into myeloid-derived suppressor cells and their roles in feto-maternal immune cross-talk. J Reprod Immunol.

[41] Kang X, Zhang X, Liu Z, Xu H, Wang T, He L, Zhao A (2016). Granulocytic myeloid-derived suppressor cells maintain feto-maternal tolerance by inducing Foxp3 expression in CD4+CD25-T cells by activation of the TGF-β/β-catenin pathway. Mol Hum Reprod.

[42] Zhang Y-H, Tian M, Tang M-X, Liu Z-Z, Liao A-H (2015). Recent Insight into the Role of the PD-1/PD-L1 Pathway in Feto-Maternal Tolerance and Pregnancy. Am J Reprod Immunol N Y N 1989.

[43] Chesneau M, Michel L, Degauque N, Brouard S (2013). Regulatory B cells and tolerance in transplantation: from animal models to human. Alloimmunity Transplant.

[44] Rolle L, Memarzadeh Tehran M, Morell-García A, Raeva Y, Schumacher A, Hartig R, Costa S-D, Jensen F, Zenclussen AC (2013). Cutting Edge: IL-10-Producing Regulatory B Cells in Early Human Pregnancy. Am J Reprod Immunol.

[45] Jensen F, Muzzio D, Soldati R, Fest S, Zenclussen AC (2013). Regulatory B10 Cells Restore Pregnancy Tolerance in a Mouse Model. Biol Reprod.

